# *ARL4C* is associated with epithelial-to-mesenchymal transition in colorectal cancer

**DOI:** 10.1186/s12885-023-10958-4

**Published:** 2023-05-26

**Authors:** Ryo Kanai, Takeshi Uehara, Takahiro Yoshizawa, Masato Kamakura, Tomoyuki Nakajima, Yasuhiro Kinugawa, Mai Iwaya, Shiho Asaka, Masato Kitazawa, Tadanobu Nagaya, Hiroyoshi Ota

**Affiliations:** 1 Department of Laboratory Medicine, Shinshu University School of Medicine, 3-1-1 Asahi, Matsumoto, Nagano, 390-8621 Japan; 2 Division of Gastroenterological, Hepato-Biliary-Pancreatic, Transplantation and Pediatric Surgery, Shinshu University School of Medicine, Matsumoto, Japan; 3 Department of Gastroenterology, Shinshu University School of Medicine, Matsumoto, Japan; 4 Department of Biomedical Laboratory Medicine, Shinshu University School of Medicine, Matsumoto, Japan

**Keywords:** ARL4C, Colorectal cancer, Tumor budding, RNA *in situ* hybridization, Colorectal adenocarcinoma

## Abstract

**Background:**

ADP-ribosylation factor-like protein 4 C (ARL4C) is a member of the ARF small GTP-binding protein subfamily. The *ARL4C* gene is highly expressed in colorectal cancer (CRC). ARL4C protein promotes cell motility, invasion, and proliferation.

**Methods:**

We investigated the characteristics of *ARL4C* by comparing its expression at the invasion front and relationships with clinicopathological data using RNAscope, a highly sensitive RNA in situ method.

**Results:**

In all cases, *ARL4C* expression was observed in cancer stromal cells and cancer cells. *ARL4C* expression in cancer cells was localized at the invasion front. In cancer stromal cells, *ARL4C* expression was significantly stronger in cases with high-grade tumor budding than in cases with low-grade tumor budding (P = 0.0002). Additionally, *ARL4C* expression was significantly increased in patients with high histological grade compared with those with low histological grade (P = 0.0227). Furthermore, *ARL4C* expression was significantly stronger in lesions with the epithelial-to-mesenchymal transition (EMT) phenotype compared with the non-EMT phenotype (P = 0.0289). In CRC cells, *ARL4C* expression was significantly stronger in cells that had the EMT phenotype compared with those with a non-EMT phenotype (P = 0.0366). *ARL4C* expression was significantly higher in cancer stromal cells than in CRC cells (P < 0.0001).

**Conclusion:**

Our analysis reinforces the possibility that *ARL4C* expression worsens the prognosis of patients with CRC. Further elucidation of the function of *ARL4C* is desired.

## Background

Colorectal cancer (CRC) is one of the leading causes of death worldwide. As Western diet and lifestyle habits are increasingly adopted globally, CRC rates are rapidly increasing. In 2018, 1.8 million people were newly diagnosed with CRC, and approximately 880,000 people died from CRC [1]. CRC ranks third among all cancers in terms of incidence and second in terms of mortality. CRC is predominantly an adenocarcinoma [2]. The adenoma-carcinoma sequence is particularly well known in CRC carcinogenesis [3]. Furthermore, chromosomal instability, microsatellite instability (MSI), and the CpG island methylator phenotype have been found to be associated with carcinogenesis [4–6]. In recent years, omics-based analyses including those of the genome, epigenome, transcriptome, and metabolome have reconfirmed and integrated concepts related to carcinogenesis, which have progressed our understanding of carcinogenic mechanisms.

Elucidation of the etiology of invasion and metastasis in CRC is progressing. In the RAS/RAF/MAPK pathway involving KRAS and BRAF, RAS activation promotes cell survival, tumor invasion, and metastasis [7]. KRAS mutation and aberrant TP53 expression regulate VEGF and VEGFR activity and promote cancer growth and migration [7]. However, much is still unknown about cancer invasion and metastasis.

ADP-ribosylation factor-like protein 4 C (ARL4C) is a member of the ARF small GTP-binding protein subfamily [8]. Simultaneous activation of the Wnt/β-catenin and EGF/RAS pathways results in *ARL4C* gene expression, leading to epithelial cell morphological changes and tubular structure formation [9]. The *ARL4C* gene is highly expressed in CRC, lung adenocarcinoma, lung squamous cell carcinoma, and tongue squamous cell carcinoma [10] [11]. ARL4C also promotes cell motility, invasion, and proliferation [11]. Hu et al. reported that *ARL4C* causes peritoneal dissemination in gastric cancer [12]. ARL4C is a potential therapeutic target because tumor growth is suppressed by siRNA against *ARL4C* [10]. Several other studies have explored ARL4C as a potential therapeutic target [13–15].

It has been reported that the appearance of the invasion front affects the prognosis of CRC [16, 17]. In this study, we investigated the characteristics of *ARL4C* by comparing its expression in the invasion front and relationships with clinicopathological data in human CRC. We utilized an RNAscope kit from Advanced Cell Diagnostics (Hayward, CA, USA) to analyze *ARL4C* mRNA expression. This in situ hybridization technique is highly sensitive with minimal background noise, and utilizes a unique double “Z-shaped” probe that targets RNA sequences spanning approximately 18–25 bases. Upon hybridization, the probe binds to amplifier probes that recognize the chromogenic label. The RNAscope method is well-suited for semi-quantitative analysis and enables precise expression level analysis.

## Methods

### Patients and materials

In total, 92 cases of CRC that were treated at Shinshu University (Matsumoto, Japan) between 2018 and 2020 were selected for this study. Among them, 14 cases were excluded because of insufficient samples available for evaluation, and 12 cases of mucinous adenocarcinoma were also excluded. Finally, 66 cases of CRC with invasion were examined.

### Histopathology and immunohistochemistry

A tissue microarray (TMA) was constructed as previously reported [18]. Briefly, the TMA was constructed from specimens fixed in 4% formaldehyde and embedded in paraffin. We collected clinicopathological data from the patients’ medical records. Two pathologists (T.U. and M.I.) re-evaluated the histological features of all specimens.

One 3-mm core centered on the invasion front was created from a sample from each patient. The 3-mm core was sufficient to assess the pathological condition of the surroundings. The captured area was carefully selected for all hematoxylin–eosin staining specimens of pre-prepared excision material. Tumor budding (TB) was carefully investigated before the region used in the TMA was chosen. TB was graded as Bd1 (0–4 buds), Bd2 (5–9 buds), and Bd3 (≥ 10 buds) [19]. Furthermore, TB grades were categorized into low-grade (Bd1) and high-grade (Bd2 and Bd3).

Immunohistochemistry was performed for E-cadherin (clone 36; dilution 1:2000; BD Biosciences, Franklin Lakes, NJ, USA) and vimentin (clone V9; dilution 1:50; Leica, Wetzlar, Germany). For antigen retrieval, sections were microwaved in 0.45% Tris/5 mM EDTA for 30 min. Detection of the primary antibodies was performed using an Envision detection system (Agilent Technologies, Santa Clara, CA, USA) in accordance with the manufacturer’s recommendations. In accordance with a previous report [18], membranous E-cadherin expression was divided into grades 0 to 3. Scores 0 and 1 were classified as E-cadherin negative, and scores 2 and 3 were classified as E-cadherin positive. For vimentin, clear positive staining in the cytoplasm of tumor cells was regarded as positive expression.

Epithelial-mesenchymal transition (EMT) phenotypes were divided into (1) non-EMT type, which was defined as E-cadherin positive and vimentin negative, (2) incomplete EMT type, which was defined as E-cadherin negative and vimentin negative or E-cadherin positive and vimentin positive, and (3) complete EMT type, which was defined as E-cadherin negative and vimentin positive, in accordance with a report by Aruga et al. [20]. The incomplete and complete EMT types were analyzed together as the EMT phenotype group and the non-EMT type was analyzed as the non-EMT phenotype group.

***ARL4C*RNA** in situ **hybridization**.

The detection of *ARL4C* mRNA was performed using an RNAscope kit (Advanced Cell Diagnostics, Hayward, CA, USA) in accordance with the manufacturer’s instructions [21]. The standard positive control (Mm-PPIB, ACD-313,902) and negative control (DapB, ACD-310,043) probes were used to ensure interpretable results. Brown punctate dots in the nucleus and/or cytoplasm indicated positive staining. *ARL4C* expression was quantified under a 20× objective lens (Olympus BX51, Tokyo, Japan) and was scored according to a five-grade scoring system. Furthermore, *ARL4C* mRNA expression was categorized into low expression (grades 0, 1+, and 2+) and high expression (grades 3 + and 4+). We analyzed the relationship between *ARL4C* expression and the clinicopathological data and prognosis of patients with CRC, with a focus on overall survival (OS).

### CIBERSORT Analysis

To investigate the relationship between *ARL4C* expression and infiltrating immune cells in CRC, we analyzed 497 cases and 17,501 genes from the CRC dataset in the Pan-Cancer Atlas of The Cancer Genome Atlas (TCGA). We excluded pTis and pT1, POLE-mutated, and MSI-H colorectal adenocarcinoma, as well as cases and genes with missing information. We used the CIBERSORT algorithm to evaluate the expression levels of 22 types of immune cells in the TCGA databases.

### Statistical analysis

Fisher’s exact test or the Wilcoxon rank-sum test were used to assess between-group differences. A P-value < 0.05 was considered significant. Spearman’s rank correlation coefficient analysis was used to assess correlations. The OS rates of CRC patients were calculated using the Kaplan–Meier method, and differences were compared using the log-rank test. All statistical analyses were performed using JMP Statistics software version 13 (JMP, Tokyo, Japan).

## Results

### ***ARL4C*** expression

**Fig. 1:**
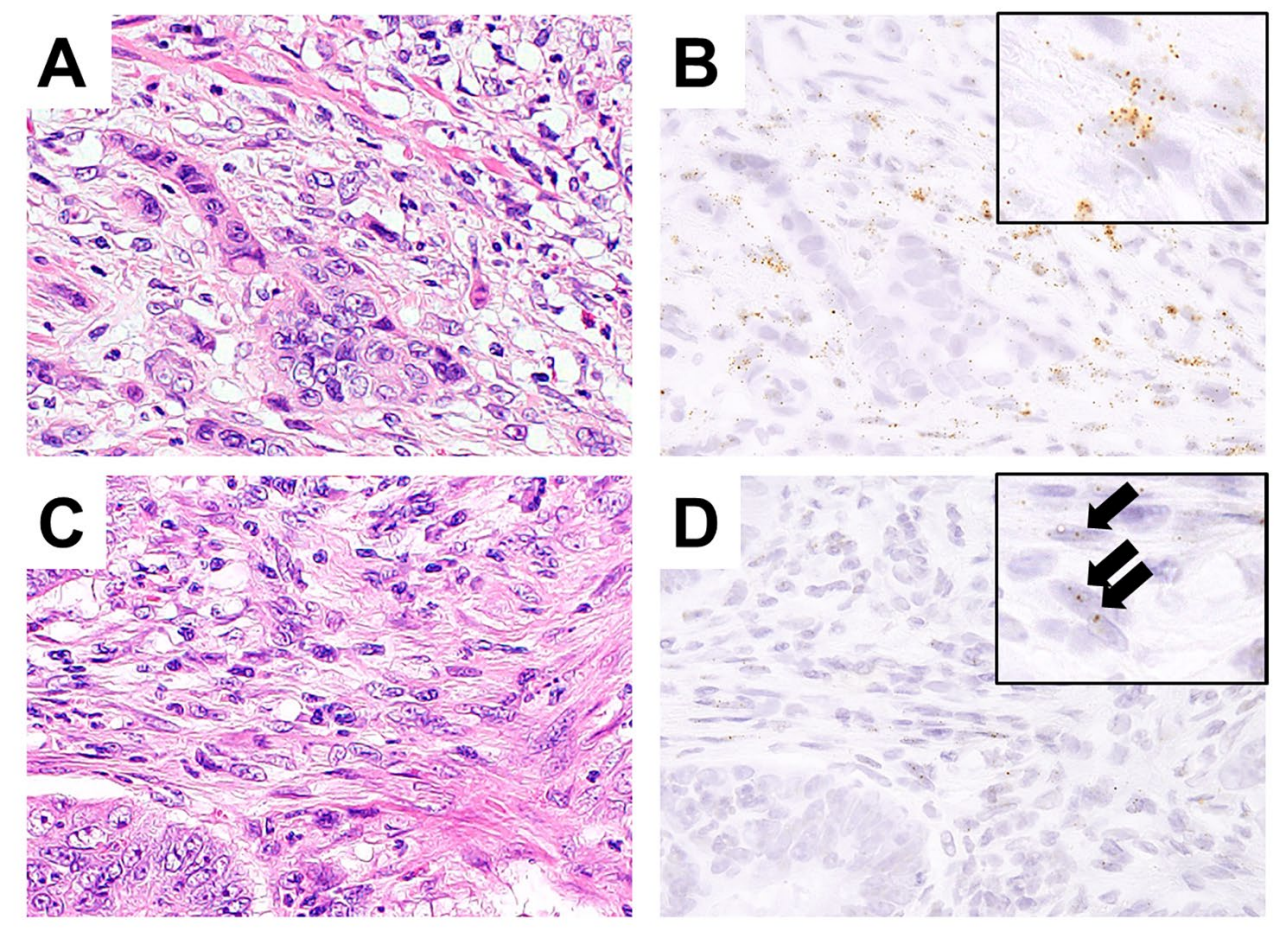
*ARL4C* expression. Representative features of higher *ARL4C* expression in cancer stromal cells (**A** and **B**). Detailed images of *ARL4C*-positive dots are shown in the insert image in B. Representative features of lower *ARL4C* expression (**C** and **D**). Detailed images of *ARL4C*-positive dots (arrows) are shown in the insert image in D. (A and C, hematoxylin eosin; B and C, *ARL4C*).

In all cases, *ARL4C* expression was observed in cancer stromal cells and cancer cells (Fig. 1). In cancer cells, *ARL4C* expression was localized at the invasion front. *ARL4C* expression was diffuse-positive in 17 cases and localized to the invasion front including TB in 49 cases. In cancer stromal cells, *ARL4C* expression were identified near cancer cells and *ARL4C* expression varied from diffuse to scattered patterns.

### Relationship between ***ARL4C*** expression and clinicopathological characteristics

**Fig. 2:**
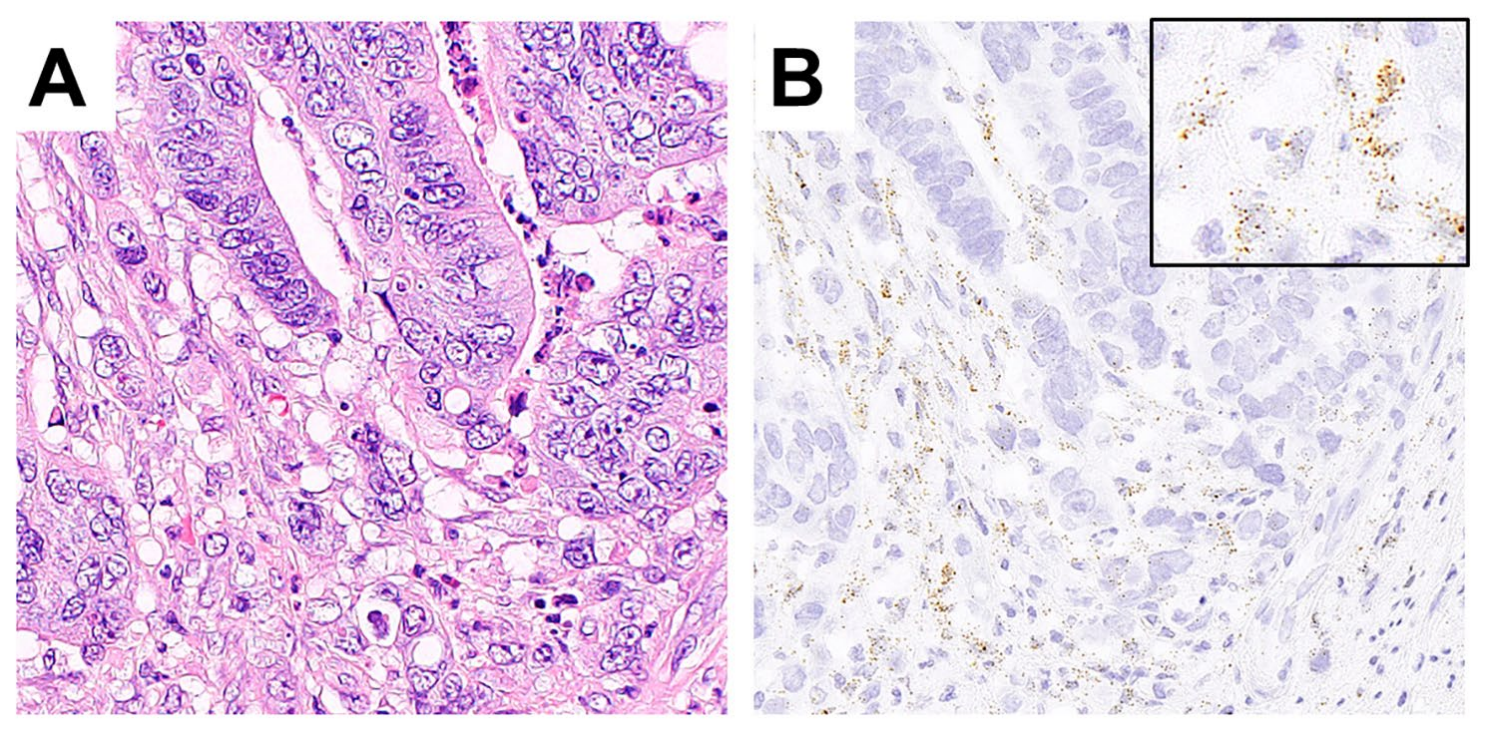
*ARL4C* expression in cancer stromal cells in the tumor budding (TB) region. Representative features of cases with higher TB grade and higher *ARL4C* expression (**A** and **B**). Detailed images of *ARL4C*-positive dots are shown in the insert image in B. (A, hematoxylin eosin; B, *ARL4C*).

Clinicopathological data are shown in Table 1. In cancer stromal cells, *ARL4C* expression was significantly stronger in cases with high-grade TB than in cases with low-grade TB (P = 0.0002) (Fig. 2). Additionally, *ARL4C* expression was significantly stronger in cases with a high histological grade than in those with a low histological grade (P = 0.0227). Furthermore, *ARL4C* expression was significantly stronger in cases with the EMT phenotype compared with those with the non-EMT phenotype (P = 0.0289).

**Table 1:** Relationships between *ARL4C* expression and clinicopathological characteristics

		*ARL4C* expression in cancer cells			*ARL4C* expression in stromal cells		
Factors	n	High (n = 52)	Low (n = 14)	P value	High (n = 54)	Low (n = 12)	P value
Age				0.1378			0.7584
≥70 years	36	31	5		30	6	
<70 years	30	21	9		24	6	
Sex				1.0000			0.5324
Male	34	27	7		29	5	
Female	32	25	7		25	7	
Histological grade				0.2182			**0.0227**
High	25	22	3		24	1	
Low	41	30	11		30	11	
Vascular invasion				0.2794			0.1246
High	51	42	9		44	7	
Low	15	10	5		10	5	
EMT				**0.0366**			**0.0289**
EMT phenotype	36	32	4		33	3	
Non-EMT phenotype	30	20	10		21	9	
Tumor budding				0.2149			**0.0002**
High-grade	43	36	7		41	2	
Low-grade	23	16	7		13	10	
TNM stage				0.5536			0.5324
I–II	32	24	8		25	7	
III–IV	34	28	6		29	5	

EMT, epithelial-to-mesenchymal transition; TNM stage, tumor, node, metastasis stage

In cancer cells, *ARL4C* expression was significantly stronger in cells with the EMT phenotype compared with those with the non-EMT phenotype (P = 0.0366).

In cancer stromal cells, there was weak positive correlation between *ARL4C* expression and TB grades (r = 0.3526, P = 0.0037). However, in cancer cells, *ARL4C* expression was not correlated with TB grades (r = 0.1730, P = 0.1647).

### Comparison of ***Arl4c*** expression between cancer stromal cells and cancer cells

**Fig. 3:**
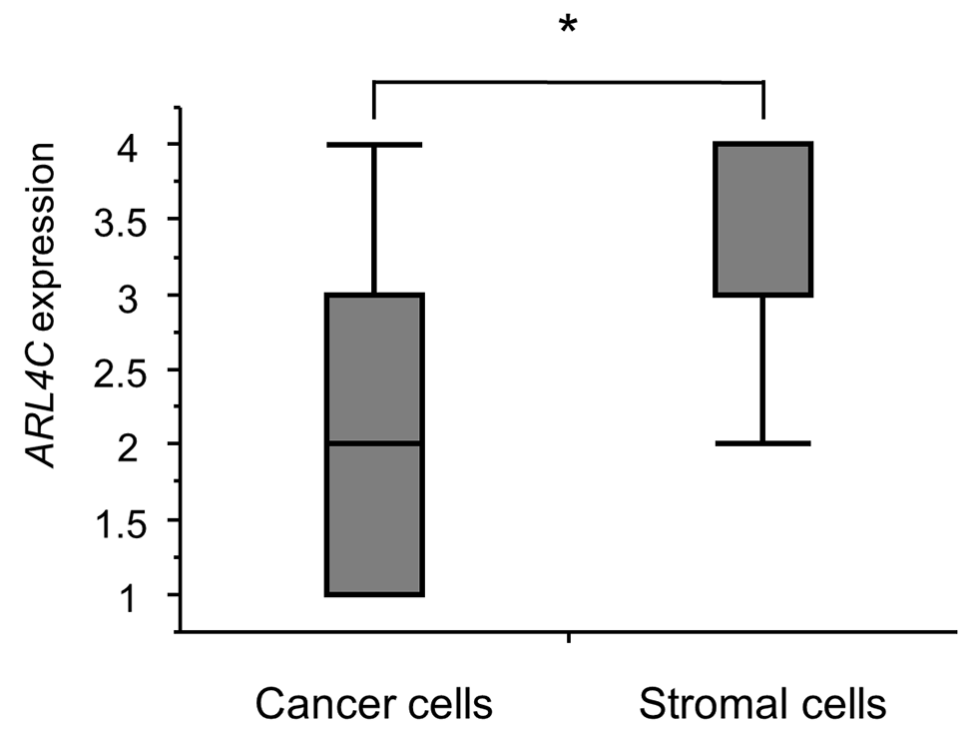
Box plot of *ARL4C* expression scores in cancer stromal cells and cancer cells. *ARL4C* scores were significantly higher in cancer stromal cells than in cancer cells (P < 0.0001)

*ARL4C* expression was significantly higher in cancer stromal cells than in cancer cells (P < 0.0001) (Fig. 3).

### Prognostic value of ***ARL4C*** in CRC

The prognostic value of *ARL4C* expression in CRC was analyzed by the Kaplan–Meier method and log-rank test. The median OS for the study patients was 24 (range; 17–34) months. A significant difference in OS was not found between CRC patients in the *ARL4C*-high expression group [median OS: 23 (range; 17.5–33.5) months] and *ARL4C*-low expression group [median OS: 32 (range; 12.75–45.5) months] (log-rank test, P = 0.6921).

### Correlation of immune cells by CIBERSORT Analysis

Our analysis revealed that *ARL4C* had weak but significant positive correlations with M2 macrophages (r = 0.413, P < 0.001) and M1 macrophages (r = 0.342, P < 0.001). However, there was no significant correlation between *ARL4C* expression and the other 20 types of immune cells evaluated by CIBERSORT. These findings suggest that *ARL4C* may play a role in the polarization of macrophages in CRC.

## Discussion

Although some prognostic involvement of *ARL4C* expression in colorectal carcinoma has been suggested, our analysis reinforces the possibility that *ARL4C* expression worsens the prognosis in CRC. Moreover, *ARL4C* expression has been identified in cancer cells in other reports, but in our study, *ARL4C* expression was identified in cancer cells as well as cancer stromal cells. Furthermore, *ARL4C* expression in the cancer stromal cells was stronger, suggesting that *ARL4C* expression in cancer stromal cells may have various effects on the tumor microenvironment. Moreover, *ARL4C* expression in cancer stromal cells was associated with poorly differentiated adenocarcinoma components and higher TB grade. Both are known to be factors that worsen a prognosis, suggesting *ARL4C* expression in cancer stromal cells is of great prognostic significance.

*ARL4C* was highly expressed in cancer cells and cancer stromal cells with the EMT phenotype. This is consistent with previous reports of other cancers [12]. *ARL4C* expression appeared to be strongest at the invasion front. Because TB grade has a strong influence on prognosis, the association between *ARL4C* expression and TB grade may have a strong influence on cancer metastasis. Further analysis of *ARL4C* in cancer cells of TB is necessary.

ARL4C expression in cancer stromal cells has not been reported by immunostaining, so *ARL4C* expression may be an RNA in situ phenomenon only. However, previous ARL4C immunostaining suggests ARL4C positivity in cancer stromal cells [22]. Recently, the pathogenesis of cancer-associated fibroblasts (CAFs) has been elucidated. TGF-β1 is an important factor in CAFs. Xie et al. reported that *ARL4C* was highly correlated with TGF-β1 signaling [23]. Silencing *ARL4C* inhibited SMAD phosphorylation, which is a downstream factor of TGF-β1 signaling. Conversely, co-expression of *ARL4C* and *TGF-β1* worsened the prognosis of gastric cancer patients.

ADP-ribosylation factor 6 (ARF6) is downstream of ARL4C and has been reported to play an important role in promoting gastric cancer EMT [24, 25]. Therefore, ARL4C may promote EMT by activating ARF6. A similar phenomenon may also occur in CRC.

Harada et al. found strong *ARL4C* expression in invasive pseudopods in pancreatic cancer [26]. Invasive pseudopods are cells closely related to EMT [27], and are usually identified in TB regions [28]. Therefore, it has been speculated that *ARL4C* expression is increased in the TB region and is involved in EMT. Our results support these conclusions.

A Gene Set Enrichment Analysis (GSEA) study showed that *ARL4C* expression was positively correlated with an EMT gene set [12]. In the same paper, Hu et al. concluded that *ARL4C* expression was a poor prognostic factor. Therefore, a detailed analysis of *ARL4C* and prognosis is desired in the future.

Silencing *ARL4C* markedly inhibited gastric cancer cell proliferation and metastasis [23]. It is strange that our study did not show a significant difference in stage, which is closely related to prognosis. The reason may be related to the small number of cases. The analysis of markers that reinforce *ARL4C*, such as *ARF6*, may also be necessary.

Our study had some limitations. This study had a relatively small sample size, which may have led to unreliable estimates. Expression analysis using cultured cells is desirable to clarify the causal relationship between *ARL4C* and prognosis.

## Conclusion

Our analysis supports the possibility that *ARL4C* expression worsens prognosis. Further elucidation of the function of *ARL4C* is desired.

## Data Availability

All data generated and analyzed during the current study are available from the corresponding author on reasonable request.
